# Effects of Three Irrigation Strategies on Gas Exchange Relationships, Plant Water Status, Yield Components and Water Productivity on Grafted Carménère Grapevines

**DOI:** 10.3389/fpls.2018.00992

**Published:** 2018-07-12

**Authors:** Mauricio Zúñiga, Samuel Ortega-Farías, Sigfredo Fuentes, Camilo Riveros-Burgos, Carlos Poblete-Echeverría

**Affiliations:** ^1^Research and Extension Center for Irrigation and Agroclimatology, Universidad de Talca, Talca, Chile; ^2^Instituto de Investigaciones Agropecuarias, INIA Carillanca, Santiago, Chile; ^3^Research Program on Adaptation of Agriculture to Climate Change (A2C2), Universidad de Talca, Talca, Chile; ^4^School of Agriculture and Food, Faculty of Veterinary and Agricultural Sciences, The University of Melbourne, Melbourne, VIC, Australia; ^5^Department of Viticulture and Oenology, Faculty of AgriSciences, Stellenbosch University, Stellenbosch, South Africa

**Keywords:** Carménère vineyards, yield parameters, deficit irrigation, plant water status, water productivity

## Abstract

In the Chilean viticultural industry, Carménère is considered an emblematic cultivar that is cultivated mainly in arid and semi-arid zones. For this reason, it is necessary to use precise irrigation scheduling for improving water use efficiency (WUE), water productivity (WP), yield and wine quality. This study evaluated the effects of three deficit irrigation strategies on gas exchange variables, WUE, WP and yield components in a drip-irrigated Carménère vineyard growing under semi-arid climatic conditions during two consecutive seasons (2011/12 and 2012/13). The irrigation strategies were applied in completely randomized design from fruit set (S) to harvest (H). The first irrigation strategy (T1) involved continuous irrigation at 100% of actual evapotranspiration (ET_a_) from S to the veraison (V) period and at 80% of ET_a_ from V to H. The second irrigation strategy (T2) involved irrigation at 50% of ET_a_ from S to H and the third one (T3) involved no-irrigation from S to V and at 30% of ET_a_ from V to H. The results indicated that there was a significant non-linear correlation between net CO_2_ assimilation (A_N_) and stomatal conductance (g_s_), which resulted in three zones of water stress (zone I = g_s_ > 0.30 mol H_2_O m^-2^s^-1^; zone II = between 0.06 and 0.30 mol H_2_O m^-2^s^-1^; and zone III = g_s_ < 0.06 mol H_2_O m^-2^s^-1^). The use of less water by T2 and T3 had a significant effect on yield components, with a reduction in the weight and diameter of grapes. A significant increase in WP (7.3 kg m^-3^) occurred in T3, which resulted in values of WUE that were significantly higher than those from T1 and T2. Also, a significant non-linear relationship between the integral water stress (SI_Ψ_) and WP (*R*^2^ = 0.74) was established. The results show that grafted Carménère vines were tolerant to water stress although differences between cultivars/genotypes still need to be evaluated.

## Introduction

“Carménère” is considered one of the most important and emblematic cultivars for the Chilean wine production. The recent reintroduction of this cultivar is exclusive to Chilean production after disappearing from Europe in the XIXth century during Phylloxera spread (Jara-Rojas et al., 2015). Carménère is usually cultivated in arid and semi-arid regions that have low to no rainfall during the growing season, making this cultivar a relevant choice for adapting viticulture to water scarcity. For this reason, it is necessary to evaluate irrigation strategies for improving water use efficiency (WUE) and water productivity (WP) (Cifre et al., 2005; Möller et al., 2006; Ruiz-Sanchez et al., 2010; Medrano et al., 2015a). One of the most implemented strategies for improving WUE and WP is the regulated deficit irrigation (RDI), which has been widely tested in fruit trees (e.g., González-Altozano and Castel, 1999; Goldhamer and Beede, 2004; Goldhamer et al., 2006; Spreer et al., 2007; Ahumada-Orellana et al., 2017) and vineyards (e.g., McCarthy et al., 2002; Girona et al., 2006; Ortega-Farias et al., 2012; Romero et al., 2015). The RDI strategy keeps water replenishment below actual vineyard evapotranspiration (ET_a_) during phenological periods when vines are less sensitive to water stress, such as the veraison period. In veraison cell division stops but cell elongation begins (Fereres et al., 2003; Basile et al., 2011). There are several studies based on the effects of RDI implementation on yield, grape quality and WUE during pre-veraison (McCarthy et al., 2002; Poni et al., 2014), post-veraison (Intrigliolo et al., 2016) or both phenological periods (Chaves et al., 2007; Basile et al., 2011). Furthermore, the effects of RDI on gas exchange variables, such as stomatal conductance (g_s_), net assimilation (A_N_) and leaf transpiration (E) have been widely studied in vineyards (e.g., Ruiz-Sanchez et al., 2010; Santesteban et al., 2011; Romero et al., 2013; Intrigliolo et al., 2016; Merli et al., 2016).

Also, g_s_, A_N_ and E are affected by environmental and specific vineyard characteristics such as atmospheric demand that varies throughout the season, soil type, cultivar, rootstock, and different viticultural practices including the use of cover crops (Medrano et al., 2015a,b). Considering all the aforementioned factors, the effects of RDI on final yield and grape quality depend first on the grapevine phenological stage at which RDI is applied and second on the severity of the stress imposed (McCarthy et al., 2002; Acevedo-Opazo et al., 2010; Romero et al., 2010). Due to the interaction of multiple site-specific factors, there is no single conclusion regarding the effects of RDI on yield parameters, WUE or WP (Intrigliolo and Castel, 2008; Uriarte et al., 2016). Therefore, it is necessary to evaluate the effect of water stress on gas exchange relationships, WUE and WP in Carménère vines specially under current water scarcity scenarios.

In order to conserve water, plants growing under a water restriction increase stomata closure which causes a decrease in the plant water potential and intensity of photosynthetic assimilation of CO_2_ (Rivero et al., 2009; Vineeth et al., 2016). Different researcher showed that g_s_ is the earliest physiological factor to be affected under mild-to-moderate water stress conditions (Flexas et al., 2002; Medrano et al., 2002; Chaves et al., 2003; Cifre et al., 2005). Thus, three phases of g_s_ responses to water stress have been previously described for mild (0.5–0.7 > g_s_ > 0.15 mol H_2_O m^-2^s^-1^), moderate (0.15 > g_s_ > 0.05 mol H_2_O m^-2^s^-1^) and severe (g_s_ < 0.05 mol H_2_O m^-2^s^-1^) water stress (Medrano et al., 2002; Cifre et al., 2005; Jara-Rojas et al., 2015). Moreover, some limitations of biochemical reactions could negatively affect A_N_ and reduce WUE for vines growing under severe water stress (Medrano et al., 2002; Cifre et al., 2005). However, obtaining accurate g_s_ measurements under field conditions can be cost prohibitive due to specialized instrumentation requirements; intensive labor needed to achieve spatial variability of g_s_, which requires qualified personnel to operate instruments and analyze data collection (Fuentes et al., 2012; Sepulveda-Reyes et al., 2016). As such, the monitoring of vine water status constitutes a suitable and affordable technique for properly achieving adequate levels of WUE and WP for sustainable grapevine production (Pellegrino et al., 2006; Acevedo-Opazo et al., 2010; Romero et al., 2013). In this sense, several studies have suggested the use of the stem water potential (Ψ_s_), which has been shown to be a more reliable and representative parameter for determining whole-plant water status. Several studies indicated that Ψ_s_ is less variable and accurate enough to detect small but statistically significant differences among irrigation treatments compared to predawn and leaf water potential measurements (Choné et al., 2001; Lampinen et al., 2001; Williams and Araujo, 2002; Romero et al., 2010; Ahumada-Orellana et al., 2017). Similarly to g_s_, vine water status responses to water stress have been reported within three thresholds: (i) mild (Ψ_s_ > -1.0 MPa), (ii) moderate (-1.0 MPa > Ψ_s_ > -1.2 MPa) and (iii) severe water (Ψ_s_ < -1.4 MPa) stress (Sibille et al., 2007; Van Leeuwen et al., 2009; Acevedo-Opazo et al., 2010). Based on these thresholds, there is no clear report relating the levels of vine water status with gas exchange responses in Carménère grapevines. Furthermore, the effects of water stress on yield, WUE and WP in Carménère grapevines also remain unevaluated. Hence, application of a correct RDI strategy for Carménère grapevines requires unified criteria between the g_s_ and Ψ_s_ levels of response to water stress; these unified criteria, in turn, would allow the acquisition of adequate results in terms of yield, WUE and WP. Thus, the aim of this study was to evaluate the effects of three irrigation strategies on the gas exchange relationships, vine water status, WUE, WP and yield components on grafted Carménère grapevines growing under Mediterranean climatic conditions. Also, the leaf gas exchange relationships were used to generated different water stress levels.

## Materials and Methods

### Study Site

The experimental trial was located in a commercial vineyard in the Talca Valley in the Maule Region of Chile (35°27,678′ LS; 71°29,951′ LW; 172 m.a.s.l.) during two consecutive seasons (2011/12 and 2012/13). The climate for this region is classified as Mediterranean semi-arid with an average daily temperature of 17.1°C and a mean annual rainfall of 679 mm (Poblete-Echeverria et al., 2012). The summer period is usually cloudless, dry and hot and; only 2.2% of the annual rainfall occurs during this period, while spring is considered wet (16% of the annual rainfall occurs during this period). The spatial variability of soil at the experimental plot was very small with effective rooting depth and water holding capacity ranging between 55–60 cm and 124–135 mm, respectively. The soil is classified as a Talca series (fine, mixed, thermic Ultic Haploxeralfs) with a clay loam texture and an average bulk density of 1.5 g cm^-3^. At the effective rooting depth, the volumetric soil water content at field capacity and the wilting point were 0.30 and 0.15 m^3^ m^-3^, respectively. The vineyard was irrigated using a single dripper per vine (4 L h^-1^). The Carménère vines were grafted on 1103 Paulsen rootstocks and planted in 2008 in a north–south orientation in rows separated by 2.5 m, and the distance between vines was 1 m. The grapevines were trained on a vertical shoot-positioned (VSP) system, with the main wire located at 1 m above ground level. The shoots were maintained in a vertical plane by two wires, with the highest one located 1.5 m above ground level.

### Weather Variables

Standard weather variables, collected from an automatic meteorological station (Adcon Telemetry, A730, Klosterneuburg, Austria) located near the experimental site (500 m), were used to calculate the reference evapotranspiration (ET_0_) over a well-irrigated grass. Air temperature (T_a_), relative humidity (RH), solar radiation (R_s_), precipitation (P_P_), wind speed (W_s_), and wind direction (W_d_) were measured at 30 min time from May 1st to April 30th during each season. The ET_a_ was calculated by adjusting the ET_0_ computed using the Penman–Monteith model by the crop coefficient (*K*_c_) corresponding to each phenological stage (Allen et al., 1998). The *K*_c_ values used in this study for Carménère vines were 0.11, 0.24, 0.61, and 0.49 for the budburst, fruit set, veraison and near-harvest periods, respectively (Jara-Rojas et al., 2015). To characterize the effects of weather conditions on grapevine phenology at the experimental site, the thermal time was calculated as growing degree days (GDD):

1 $$\text{GDD}=\sum_{\text{i}=1}^{\text{n}}\frac{\text{Tmax}+\text{Tmin}}{2}-{\text{T}}_{\text{base}}$$

where T_max_ and T_min_ are daily maximum and minimum temperatures (°C), respectively, and T_base_ is the base temperature (10°C).

### Experimental Design

The experimental design consisted of the implementation of three irrigation strategies with four replicates per treatment in a completely randomized design (CRD). This design was selected because the experiment was stablished in a vineyard block with uniform soil and vine canopy. In the experimental site, there were not significant differences among treatments for trunk diameter with values ranging between 41.4 and 41.6 mm (**Table 1**). The experimental unit corresponded to six adjacent vines. Irrigation was maintained at 100% of ET_a_ from budbreak (B) to fruit set (S) during both seasons. The first irrigation strategy (T1) involved continuous irrigation at 100% of ET_a_ from S to veraison (V) and at 80% of ET_a_ from V to harvest (H); the second irrigation strategy (T2) involved irrigation at 50% of ET_a_ from S to H; and the third irrigation strategy (T3) involved no-irrigation from S to V and at 30% of ET_a_ from V to H (**Table 1**).

**Table 1:** Water application as a percentage of actual evapotranspiration (ET_a_).

Irrigation strategies	S-V (%)	V-H (%)	TD (mm)
T1	100	80	41.4
T2	50	50	41.4
T3	0	30	41.6
Significance	–	–	0.94

*Also, trunk diameter (TD) is included as a reference. S, setting; V, veraison; H, harvest. Values followed by the same letter are not significantly different (Tuckey p ≤ 0.05).*

### Plant Water Status and Leaf Gas Exchange Measurements

To evaluate vine water status, Ψ_s_ was measured weekly around midday (between 13 and 15 h) (Coordinated Universal Time UTC-3) using a pressure chamber (PMS Instrument Company, Model 1000 Pressure Chamber Instrument). For this purpose, two mature healthy non-damaged leaves per replicate were selected and measured (Choné et al., 2001). Each selected leaf (still attached to the vine) was wrapped with plastic transparent film and covered with aluminum foil at least 2 h before being measured. By reducing leaf transpiration to near zero (plastic) and avoiding overheating (aluminum), this process allowed the water potential to equilibrate between the leaf and xylem of the plant.

To determine the accumulative effects of water deficit, integral water stress (SI_Ψ_) was calculated as follows (Myers, 1988):

1 $${\text{SI}}_{\text{Ψ}}=\sum ({\bar{\text{Ψ}}}_{\text{s}}-\text{c})\text{n}$$

where Ψ_s_ is the average of stem water potential for any interval (MPa), *c* is the maximum stem water potential value (-0.4 MPa for both seasons) during the whole period and *n* is the number of days between measurements.

Measurements of photosynthetically active radiation (PAR), net CO_2_ assimilation (A_N_), stomatal conductance (g_s_), and leaf transpiration (E) were measured in parallel with Ψ_s_ on the same vines using a portable infrared gas analyzer (LI-6400, LI-COR Inc., Lincoln, Nebraska, United States). For this purpose, another two mature healthy fully expanded and sunlit leaves were selected. During measurements, the leaf chamber temperature block was maintained between 25 and 32°C, which was within the range of air temperatures of the measurement days. The molar air flow rate inside the leaf chamber was set to 500 μmol mol^-1^. All measurements were taken at a reference CO_2_ concentration similar to that of the environment at the time of measurements (380–400 μmol mol^-1^) and with a natural saturating photosynthetic photon flux that ensured leaves received more than 1,000 μmol m^-2^s^-1^, as the leaf angle at the time of measurement was preserved (no external light source was used in this study) (Poni et al., 2014; Jara-Rojas et al., 2015). The intrinsic WUE (WUE_i_ = A_N_/g_s_), instantaneous WUE (WUE = A_N_/E), A_N_/A_Nmax_, g_s_/g_smax_ and E/E_max_ ratios were estimated with the aim of standardizing the degree of change in comparison to the maximum values (A_Nmax,_ g_smax_, and E_max_) obtained from all treatments during each season.

### Yield Components and Soluble Solids

The total yield (Yield; kg plant^-1^) was determined from the four central vines of each experimental unit, which were completely harvested at the same time as were those commercially harvested. The number of clusters (N_clusters) were registered for each vine harvested. A sample of 30 clusters from each replicate was selected to determine cluster weight (W_clusters), cluster volume (V_clusters) and the number of grapes per cluster (N_grapes). A sample of 100 randomized grapes per replicate was taken to determine grape weight (W_grapes) and grape diameter (D_grapes) (Acevedo-Opazo et al., 2010; Romero et al., 2015).

### Statistical Analysis

A regression analysis was used to evaluate the effects of the different levels of vine water stress on the degree of association between the studied variables (A_N_/A_Nmax_ vs g_s_, Ψ_s_ vs g_s_, WUEi vs g_s_, WUE vs g_s_, and WP vs SI_Ψ_). Additionally, the Root Mean Square Error (RMSE) and the Sum of the Squared Error (SSE) were calculated.

A piecewise linear regression (PLR) was performed to obtain the threshold values of the g_s_ responses to water stress in the A_N_/A_Nmax_ vs g_s_ relationship (Toms and Lesperance, 2003; Malash and El-Khaiary, 2010). In this case, the PLR method was used to evaluate the existence of abrupt changes in slope (or the ratio of A_N_/A_Nmax_ to g_s_).

In addition, principal component analysis (PCA) was used to obtain a hierarchy of the variables analyzed to find patterns in the data and to classify any combination of variables that could explain the effects of irrigation treatments on SI_Ψ_, WUE, WUE_i_, WP and yield components. Finally, data for each parameter were obtained for each replicate per treatment and were subjected to two-way analysis of variance (ANOVA) to assess the influence of each irrigation strategy and season on the physiological and yield parameters. The variables analyzed for comparative purposes within treatments were Ψ_s_, A_N_/A_Nmax_, g_s_/g_smax_, E/E_max_, WUE, WUE_i_, Yield, N_clusters, W_clusters, V_clusters, N_grapes, W_grapes, and D_grapes. The seasonality effects were estimated as “treatment × season” interactions. ANOVA was performed using the student version of the statistical software Infostat (National University of Cordoba, Argentina).

## Results

### Weather Conditions and Total Water Application

In general, the atmospheric conditions during both seasons were dry and hot and without significant rainfall from S to H specifically for 2011/12 (**Table 2**), which is consistent with the climatic description for this region. There was only 66 mm of rainfall during the 2012/13 season, with 77% concentrated during the S-V period. Additionally, the 2012/13 season had the highest amount of rainfall during the B-S period (95.2 mm). The mean air temperature ranged between 10 and 24°C, while the VPD was between 0.2 and 1.7 kPa. The maximum and minimum average temperatures were between 35–36 and 2–3°C, respectively. The highest atmospheric demand occurred near the V period with values of T_a_ between 15 and 24°C and VPD between 0.4 and 1.7 kPa. From B to H, the accumulated values of ET_0_ were 925 and 880 mm while those of the GDD were 1,547 and 1,472°C for the 2011/12 and 2012/13 growing seasons, respectively.

**Table 2:** Mean values of climate variable and water requirements for the main phenological stages of a drip-irrigated Carménère vineyard.

Season/Phenological stages		LF-B	B-S	S-V	V-H	Season
	VPD (kPa)		0.6	1.1	0.7	0.92
	T_a_ (°C)	8.2	14.9	20.5	17.5	13.4
2011/12	ET_0_ (mm)		273.1	312.5	339.7	925
	ET_a_ (mm)		31.4	73.7	207.2	312
	Pp (mm)	449.4	8.4	0	9.4	467
	GDD (°C)	48	317.7	558.4	626.1	1,547
	VPD (kPa)		0.7	1.03	0.8	0.85
	T_a_ (°C)	9.1	15.5	19.9	17	13.6
2012/13	ET_0_ (mm)		264.5	287.4	328.2	880
	ET_a_ (mm)		30.6	71.1	196.6	298
	Pp (mm)	416.4	95.2	50.8	15.2	578
	GDD (°Cd)	59	339.4	494	580.5	1,473

*LF, leaf fall; B, Budburst; S, setting; V, veraison; H, harvest; VPD, vapor pressure deficit; T_a_, air temperature; Pp, rainfall; ET_0_, reference evapotranspiration; ETa, actual evapotranspiration; GDD, growing degree days.*

The total water applied ranged from 688 m^3^ ha^-1^ for T3 during the 2012/13 season to 2,692 m^3^ ha^-1^ for T1 during the 2011/12 season (**Table 3**). For the two studied seasons, T3 received irrigation only during the V-H period. The maximum water application was observed during V-H period which presented the higher values of ET_0_ and K_c_. The highest and lowest values of vine water status were observed during S-V and H-V periods, respectively. For the both seasons, values of Ψ_s_ for T1 ranged between -0.63 and -1.01 MPa while those for T3 were between -0.76 and -1.53 MPa, respectively (**Tables 4**–**6**).

**Table 3:** Water application (m^3^ ha^-1^) from setting (S) to veraison (V) and V to harvest (H) for a drip-irrigated Carménère vineyard.

	Growing seasons					
	2011/12			2012/13		
Treatments	S-V	V-H	Total	S-V	V-H	Total
T1	1,061	1,631	2,692	1,008	1,279	2,287
T2	526	1,036	1,562	509	983	1,491
T3	0	725	725	0	688	688

### Physiological Relationships

**FIGURE 1:**
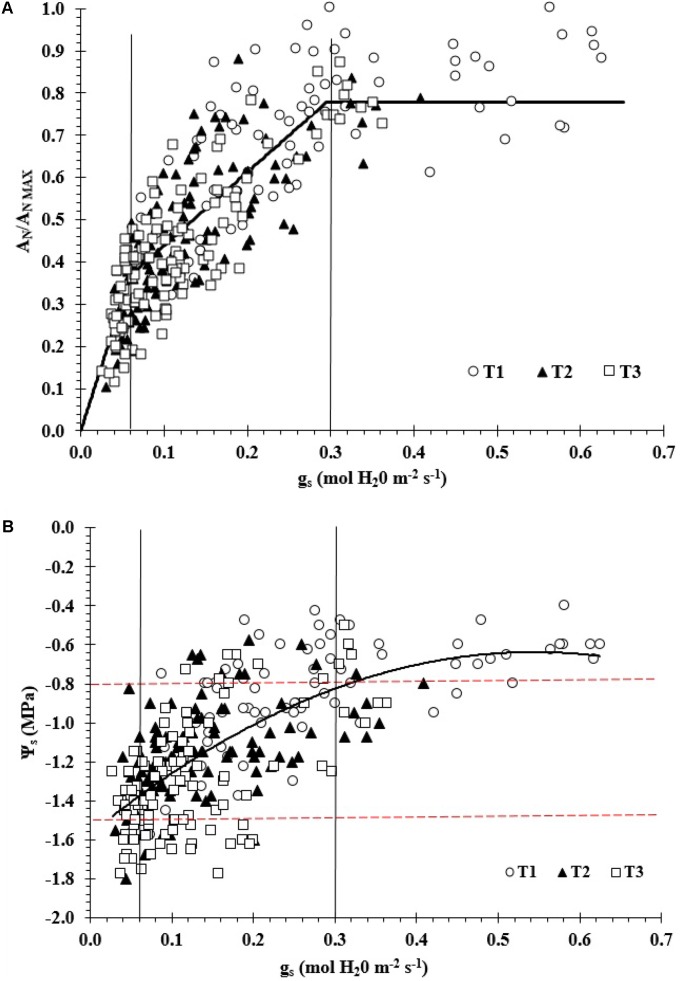
**(A)** Relationship between A_N_/A_Nmax_ ratio versus stomatal conductance (g_s_) vineyard (A_N_/A_Nmax_ = 1.02 + 0.25ln(g_s_); *R*^2^ = 0.72) and **(B)** relationship between midday stem water potential (Ψ_s_) versus g_s_ in a drip-irrigated Carménère vineyard (Ψ_s_ = –3.157g_s_^2^+3.43g_s_–1.571; *R*^2^ = 0.5). A_N_ and A_Nmax_ are actual and maximum values of net CO_2_ assimilation, respectively.

**Figure 1A** shows a significant non-linear relationship between A_N_/A_Nmax_ vs g_s_ for the overall data set (*R*^2^ = 0.72; RMSE = 0.11 (dimensionless); SEE = 0.17 (dimensionless); and *p*-value < 0.01; *n* = 304). In this study, the measured values of PAR, A_N_, g_s_, and E near noon ranged between 1,100 and 1,900 μmol m^-2^s^-1^, 1.4–19.0 μmol CO_2_ m^-2^s^-1^, 0.03–0.63 mol H_2_O m^-2^s^-1^, and 0.8–11.8 mmol m^-2^s^-1^, respectively. The maximum measured values of A_N_, g_s_, and E for the 2011/12 season were 14 μmol CO_2_ m^-2^s^-1^, 0.36 mol H_2_O m^-2^s^-1^ and 9.69 mmol m^-2^s^-1^ while those for the 2012/13 season were 19 μmol CO_2_ m^-2^s^-1^, 0.63 mol H_2_O m^-2^s^-1^ and 11.75 mmol m^-2^s^-1^, respectively.

Additionally, the piecewise linear regression analysis revealed that there were three main zones: g_s_ > 0.30 (zone I; A_N_/A_Nmax_ = 0.74), 0.06 < g_s_ < 0.30 (zone II; A_N_/A_Nmax_ = 0.26 + 1.74g_s_) and g_s_ < 0.06 mol H_2_O m^-2^s^-1^ (zone III; A_N_/A_Nmax_ = 6.14g_s_) (**Figure 1A**). In zone I, A_N_/A_Nmax_ was not significantly affected by changes in g_s_ and thus presenting a clear plateau (*R*^2^ = 0.10). For zones II and III, the A_N_/A_Nmax_ ratio decreased significantly when g_s_ value diminished, the *R*^2^ values were 0.22 and 0.26, respectively. The estimated slopes were 6.1 and 1.7 per mol H_2_O m^-2^s^-1^ for zones II and III, respectively. For zone I, the mean value of A_N_/A_Nmax_ was 0.81 (A_N_ = 13.4 μmol CO_2_ m^-2^s^-1^) when g_s_ decreased from 0.63 to 0.3 mol H_2_O m^-2^s^-1^. The A_N_/A_Nmax_ values ranged between 0.18 and 1.0 (A_N_ between 3.0 and 16.6 μmol CO_2_ m^-2^s^-1^) and 0.10–0.45 (A_N_ between 1.7 and 7.4 μmol CO_2_ m^-2^s^-1^) for zones II and III, respectively.

There was also a significantly non-linear relationship between Ψ_s_ and g_s_ (*R*^2^ = 0.5; RMSE = 0.09 mol H_2_O m^-2^s^-1^; SEE = 0.07 mol H_2_O m^-2^s^-1^; *p*-value < 0.01) (**Figure 1B**). In this case, the obtained threshold values of g_s_ from the previous piecewise linear regression analysis (0.30 and 0.06 mol H_2_O m^-2^s^-1^) were related to different levels of Ψ_s_. In this regard, the estimated Ψ_s_ thresholds were Ψ_s_ > -0.8 MPa (zone I), -0.8 > Ψ_s_ > -1.5 MPa (zone II), and Ψ_s_ < -1.5 MPa (zone III).

**FIGURE 2:**
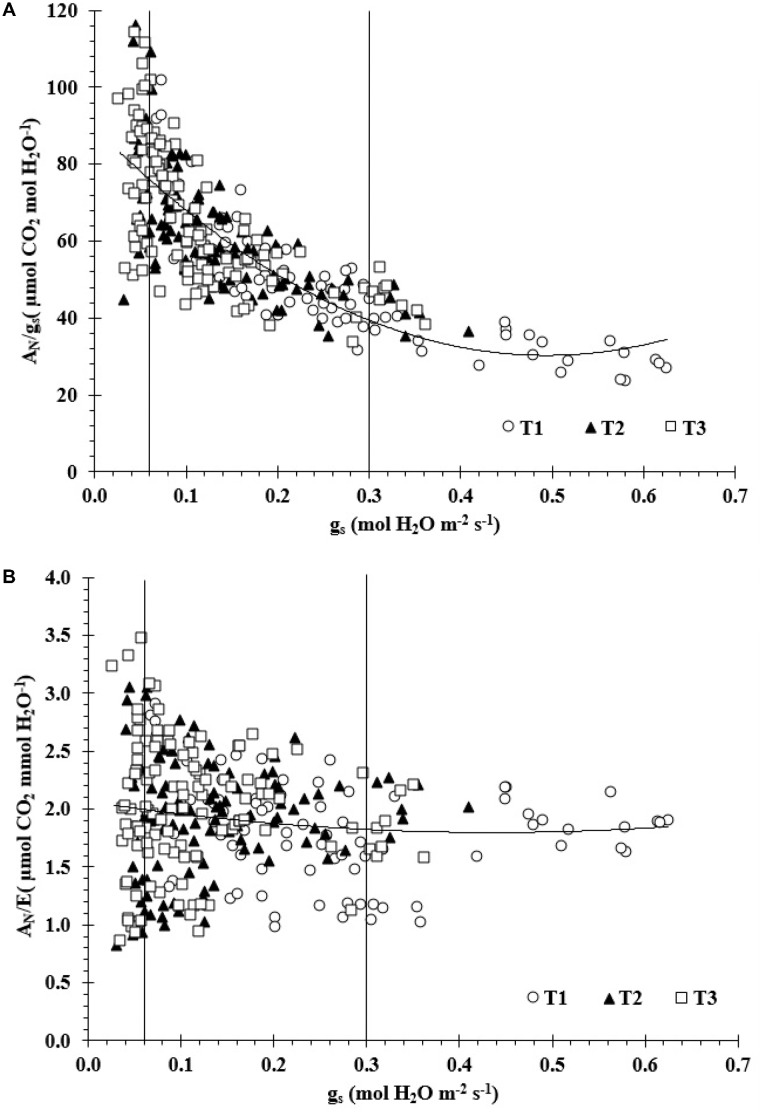
**(A)** The relationships between intrinsic water use efficiency versus stomatal conductance (g_s_) (A_N_/g_s_ = 242.81g_s_^2^–239.68g_s_+89.497; *R*^2^ = 0.62) and **(B)** between water use efficiency versus g_s_ (A_N_/E = 1.4086g_s_^2^–1.2237g_s_+2.0654; *R*^2^ = 0.02).

**Figure 2A** indicates that there were significant linear correlations between WUEi and g_s_ for Zone I and II with *R*^2^ values of 0.67 and 0.51, respectively. A large scattering of data was observed in the Zone III, which led to a not statistical significant relationship. Values of WUEi increased from 23.4 to 109.5 μmol CO_2_/mol H_2_O m^-2^s^-1^ as g_s_ diminished from 0.06 to 0.63 mol H_2_O m^-2^s^-1^ (Zone I and II). Furthermore, there was not a statistical significant relationship between WUE vs g_s_ for the three zones (**Figure 2B**).

### PCA

**FIGURE 3:**
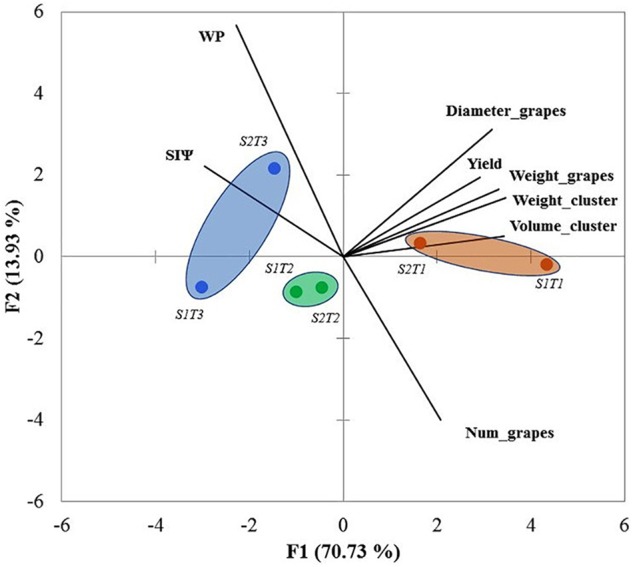
Principal component analysis (PCA) considering all data sets for the growing seasons 2011/2012 and 2012/2013 (cv. Carménère). SI_Ψ_, integral water stress; WP, water productivity; Diameter_grape, diameter of grape; Weight_cluster, weight of cluster; Weight_grape, weight of grape; Volume_cluster, volume of cluster; Num_grapes, number of grapes; S1 = season 2011/12 and S2 = season 2012/13. T1, T2, and T3 are the irrigation treatments.

Principal component 1 (PC1) accounted for 70.73% of the variability; principal component 2 (PC2), 13.93%. Therefore, the PCA presented in **Figure 3** represents a total of 84.66% of the data variability. PC1 is positively related to the yield component variables (D_grapes, W_clusters, W_grape, Yield, and V_clusters, as well as N_grapes, albeit at a small percentage) that were best associated with T1. On the other hand, the PC2 was correlated with variables influenced positively by water stress (WP and SI_Ψ_). Therefore, PC2 was associated mainly with T2 and T3.

### Water Status Dynamics and Physiological Trends

**Tables 4**–**6** show the effects of three levels of water applications on physiological variables during the S-V, V, and V-H periods, respectively. Also, Supplementary Tables S1–S3 indicate a summary of analysis of variance (ANOVA) expressed on the mean square and degree of freedom (df) for physiological variables from setting (S) to veraison (V) period of a dripirrigated Carménère vineyard. For the S-V period, significant interactions between irrigation treatments and seasons were observed for Ψ_s_, A_N_/A_Nmax_, E/E_max_, and WUE_i_. Results indicated that values of Ψ_s_, A_N_/A_Nmax_, and E/E_max_ for the 2011/12 were significantly lower than those for 2012/13. Also, there was a significant effect of water stress on vine water status and gas exchange where the highest values of Ψ_s_, A_N_/A_Nmax_, g_s_/g_smax_, and E/E_max_ were observed in T1. For this treatment, mean values of Ψ_s_, A_N_ g_s_, and E for T1 were -0.70 MPa, 12.4 μmol CO_2_ m^-2^s^-1^, 0.28 mol H_2_O m^-2^s^-1^ and 8.15 mmol m^-2^s^-1^, respectively, while those for T3 were -1.5 MPa, 6.1 μmol CO2 m^-2^s^-1^, 0.09 mol H_2_O m^-2^s^-1^ and 3.0 mmol m^-2^s^-1^, respectively.

**Table 4:** Effect of three levels of water application on physiological variables from setting (S) to veraison (V) period of a drip-irrigated Carménère vineyard.

	Ψ_s_ (MPa)		A_N_/A_Nmax_		g_s_/g_smax_		E/E_max_		WUE_i_		WUE	
**Treatments**												
T1	-0.70 a		0.75 a		0.56 a		0.76 a		47.49 b		1.5	
T2	-0.93 b		0.57 b		0.35 b		0.61 b		55.79 ab		1.5	
T3	-1.02 b		0.54 b		0.33 b		0.57 b		58.35 a		1.5	
**Season**												
2011/12	-1.06 b		0.52 b		0.37 b		0.47 b		58.85 a		1.3 b	
2012/13	-0.71 a		0.72 a		0.45 a		0.82 a		48.91 b		1.7 a	
**Treatments x**	2011/12	2012/13	2011/12	2012/13	2011/12	2012/13	2011/12	2012/13	2011/12	2012/13	2011/12	2012/13
**Seasons**												
T1	-0.78 a	-0.63 a	0.76 a	0.74 a	0.65 a	0.50 ab	0.70 a	0.90 a	45.6 b	49.3 b	1.3	1.8
T2	-1.12 b	-0.73 a	0.43 b	0.77 a	0.26 cd	0.46 abc	0.39 b	0.82 a	60.9 ab	50.7 b	1.3	1.7
T3	-1.27 b	-0.76 a	0.36 b	0.66 a	0.21 d	0.40 bcd	0.32 b	0.75 a	70.0 a	46.7 b	1.4	1.7
Treatments	<0.001		<0.001		<0.001		<0.001		<0.001		0.8415	
Season	<0.001		<0.001		0.0801		<0.001		0.0083		<0.001	
Treatments x Season	0.005		0.002		<0.001		<0.001		0.0142		0.6796	

*Values followed by the same letter are not significantly different (Tuckey p ≤ 0.05). Ψ_s_, midday stem water potential; A_N_, net CO_2_ assimilation; A_Nmax_, maximum net CO_2_ assimilation value (14 and 19 μmol CO_2_ m^-2^s^-1^ for 2011/12 and 2012/13, respectively); g_s_, stomatal conductance; g_smax_, maximum stomatal conductance value (0.36 and 0.63 mol H_2_O m^-2^s^-1^ for 2011/12 and 2012/13, respectively), E, transpiration; E_max_, maximum transpiration value (9.69 and 11.75 mmol m^-2^s^-1^ for 2011/12 and 2012/13, respectively); WUE_i_, intrinsic water-use efficiency; WUE, water-use efficiency.*

**Table 5:** Effect of three levels of water application on physiological variables during veraison (V) period of a drip-irrigated Carménère vineyard.

	Ψ_s_ (MPa)		A_N_/A_Nmax_		g_s_/g_smax_		E/E_max_		WUE_i_		WUE	
**Treatments**												
T1	-0.95 a		0.75 a		0.63 a		0.67 a		45.04 c		1.81 b	
T2	-1.16 b		0.58 b		0.38 b		0.48 b		55.91 b		1.90 ab	
T3	-1.50 c		0.37 c		0.18 c		0.28 c		71.21 a		2.06 a	
**Season**												
2011/12	-1.19		0.52 b		0.36		0.39 b		64.71 a		1.64 a	
2012/13	-1.22		0.62 a		0.43		0.56 a		50.06 b		1.21 b	
**Treatments x**	2011/12	2012/13	2011/12	2012/13	2011/12	2012/13	2011/12	2012/13	2011/12	2012/13	2011/12	2012/13
**Seasons**												
T1	-1.01	-0.89	0.72	0.79	0.58	0.68	0.56	0.77	52.26	37.81	1.58	2.03
T2	-1.03	-1.29	0.55	0.62	0.40	0.40	0.41	0.55	61.67	50.15	1.59	2.22
T3	-1.47	-1.53	0.29	0.44	0.10	0.20	0.20	0.36	80.19	62.22	1.75	2.38
Treatments	<0.001		<0.001		<0.001		<0.001		<0.001		0.0459	
Season	0.5404		0.0029		0.0632		<0.001		<0.001		<0.001	
Treatments x Season	0.234		0.4796		0.8088		0.7355		0.6544		0.5897	

*Values followed by the same letter are not significantly different (Tuckey p ≤ 0.05). Ψ_s_, midday stem water potential; A_N_, net CO_2_ assimilation; A_Nmax_, maximum net CO_2_ assimilation value (14 and 19 μmol CO_2_ m^-2^s^-1^ for 2011/12 and 2012/13, respectively); g_s_, stomatal conductance; g_smax_, maximum stomatal conductance value (0.36 and 0.63 mol H_2_O m^-2^s^-1^ for 2011/12 and 2012/13, respectively); E, transpiration; E_max_, maximum transpiration value (9.69 and 11.75 mmol m^-2^s^-1^ for 2011/12 and 2012/13, respectively); WUE_i_, intrinsic water-use efficiency; WUE, water-use efficiency.*

**Table 6:** Effect of three levels of water application on physiological variables from veraison (V) to harvest (H) period on a Drip-irrigated Carménère vineyard.

	Ψ_s_ (MPa)		A_N_/A_Nmax_		g_s_/g_smax_		E/E_max_		WUE_i_		WUE	
**Treatments**			
T1	-0.87 a		0.64 a		0.48 a		0.52 a		51.97 b		2.02 b	
T2	-1.11 b		0.49 b		0.32 b		0.34 b		61.01 b		2.11 b	
T3	-1.30 c		0.38 c		0.18 c		0.25 c		76.33 a		2.37 a	
**Season**			
2011/12	-1.01 a		0.59 a		0.36a		0.42 a		72.21 a		2.22	
2012/13	-1.18 b		0.42 b		0.29b		0.32 b		54.01 b		2.11	
**Treatments**	2011/12	2012/13	2011/12	2012/13	2011/12	2012/13	2011/12	2012/13	2011/12	2012/13	2011/12	2012/13
**x season**			
T1	0.87 a	0.87 a	0.68 a	0.61 a	0.46 a	0.51 a	0.39 b	0.64 a	61.83	42.11	2.08	1.95
T2	0.94 a	1.29 b	0.63 a	0.36 bc	0.43 a	0.21 b	0.34 bc	0.35 bc	65.94	56.09	2.13	2.10
T3	1.38 b	1.21 b	0.47 b	0.30 c	0.20 b	0.17 b	0.23 c	0.28 c	88.84	63.83	2.45	2.30
Treatments	<0.001		<0.001		<0.001		<0.001		<0.001		0.005	
Season	<0.001		<0.001		0.065		0.002		<0.001		0.247	
Treatments x season	0.0091		0.0201		0.014		0.003		0.265		0.836	

*Values followed by the same letter are not significantly different (Tuckey p ≤ 0.05). Ψ_s_, midday stem water potential; A_N_, net CO_2_ assimilation; A_Nmax_, maximum net CO_2_ assimilation value (14 and 19 μmol CO_2_ m^-2^s^-1^ for 2011/12 and 2012/13, respectively); g_s_, stomatal conductance; g_smax_, maximum stomatal conductance value (0.36 and 0.63 mol H_2_O m^-2^s^-1^ for 2011/12 and 2012/13, respectively); E, transpiration; E_max_, maximum transpiration value (9.69 mmol m^-2^s^-1^ for 2011/12 and 11.75 mmol m^-2^s^-1^ for 2012/13); WUE_i_, intrinsic water-use efficiency; WUE, water-use efficiency.*

On the other hand, regarding T3 in the 2011/12 season, the WUE_i_ showed a significantly higher value than the other treatments with almost all interactions except with T2 for the 2011/12 season. Finally, the WUE showed significant differences only between years, and the value (1.7 mmol CO_2_ mol H_2_O^-1^) was higher in the 2012/13 study season than in the 2011/12 study season.

For the V period, the mean values of Ψ_s_, A_N_/A_Nmax_, g_s_/g_smax_, and E/E_max_ for T1 were significantly higher than those observed for T2 and T3 (**Table 5**). In this case, mean values of Ψ_s_, A_N_ g_s_, and E for T1 were -0.95 MPa, 12.4 μmol CO_2_ m^-2^s^-1^, 0.31 mol H_2_O m^-2^s^-1^ and 7.2 mmol m^-2^s^-1^while those for T3 were -1.5 MPa, 6.1 μmol CO_2_ m^-2^s^-1^, 0.09 mol H_2_O m^-2^s^-1^ and 3.0 mmol m^-2^s^-1^, respectively. For the same period, the mean values of WUE_i_ (71.21 μmol CO_2_ m^-2^s^-1^/mol H_2_O m^-2^s^-1^) and WUE (2.06 mmol CO_2_/mol H_2_O^-1^) for T3 were significantly higher than those for the other treatments. Also, the A_N_/A_Nmax_ (0.62) and E/E_max_ (0.56) ratios for the 2012/13 were significantly higher than those for the 2011/12 growing season; however, the lowest mean values of WUE_i_ and WUE were observed during the 2012/13 growing season (**Table 5**).

For the V-H period, significant interactive effects of irrigation treatments and season were observed for Ψ_s_, A_N_/A_Nmax_, g_s_/g_smax_, and E/E_max_. For these parameters, T3 showed significantly lower mean values in comparison with T1 during both study seasons (**Table 6**). Results indicate that mean values of Ψ_s_, A_N_, g_s_, and E were significantly reduced by water stress from -0.87 to -1.3 MPa, 10.6–6.1 μmol CO_2_ m^-2^s^-1^, 0.24–0.09 mol H_2_O m^-2^s^-1^, and 5.7–2.7 mmol m^-2^s^-1^, respectively. In addition, mean values of WUE_i_ and WUE for T3 were significantly higher than those for T1 and T2 and significantly higher during the 2011/12 than those during the 2012/13 season.

The results showed that the values of WUE_i_ for T1 ranged between 23.4 and 73.3 μmol CO_2_/mol H_2_O m^-2^s^-1^, for T2 ranged between 41.6 and 92.7 mol CO_2_/mol H_2_O m^-2^s^-1^, and for T3 ranged between 33.3 and 114.1 mol CO_2_/mol H_2_O m^-2^s^-1^. The WUE values for T1, T2, and T3 ranged from 0.98 to 2.9, 0.94 to 2.85, and 0.86 to 3.5 μmol CO_2_/mol H_2_O m^-2^s^-1^, respectively.

### Yield Components, Water Productivity, and Integral Water Stress Effects

The results showed that there were significant differences among the irrigation strategies for yield, with the highest mean value observed for T1 (2.49 kg plant^-1^) followed by T2 (1.64 kg plant^-1^) and T3 (1.58 kg plant^-1^), of which the last two did not show differences between them (**Table 7**). In this case, there were not significant difference, between treatments or between seasons for N_clusters and N_grapes with mean values ranging between 14.90–16.90 and 92.29–101.03, respectively. W_clusters and V_ clusters showed significantly higher mean values for T1 (160.02 gr and 165.13 mL, respectively) compared to T2 (123.85 gr and 125.81 mL, respectively) and T3 (120.40 gr and 118.98 mL, respectively); the last two did not show significant differences between them. Significant interactive effects of irrigation treatments and season were observed for W_grapes and D_grapes. T1 presented the highest mean values, especially during the 2011/2012 season, compared to T2 and T3. For both studied seasons, T2 and T3 did not present significant differences between them (**Table 7**).

**Table 7:** Analysis of yield components, water productivity and integral water stress for both seasons studied.

	yield		N-cluster		N_Grapes		W_Cluster		V_Cluster		W_ Grape		D_Grape		WP		SI_Ψ_	
**Treatments**																		
T1	2.49 a		16.90		101.03		160.02 a		165.13 a		1.81		13.61		4.06 b		48.09 c	
T2	1.64 b		15.02		93.35		123.85 b		125.81 b		1.47		12.48		5.11 b		69.78 b	
T3	1.58 b		14.91		92.29		120.40 b		118.98 b		1.39		12.26		7.3 a		102.91 a	
**Season**																		
2011/12	1.81		15.44		100.74		133.33		140.07		1.57		12.56		5.17 b		74.23	
2012/13	1.99		15.48		90.37		136.18		133.21		1.53		13		6.37 a		72.95	
**Treatments x**	2011/12	2012/13	2011/12	2012/13	2011/12	2012/13	2011/12	2012/13	2011/12	2012/13	2011/12	2012/13	2011/12	2012/13	2011/12	2012/13	2011/12	2012/13
**Seasons**																		
T1	2.34	2.65	16.13	17.75	111.15	90.90	169.93	150.11	179.00	141.25	2.03 a	1.58 ab	14.05 a	13.17 a	3.48	4.65	51.40	44.78
T2	1.71	1.56	16.31	13.63	91.80	94.90	118.70	129.00	121.00	130.63	1.47 b	1.46 b	12.18 b	12.78 ab	4.38	4.18	67.35	72.20
T3	1.39	1.77	13.88	15.06	99.28	85.30	129.43	111.38	110.20	127.65	1.22 b	1.56 b	11.45 b	13.07 ab	7.65	10.28	103.95	101.88
Treatments	<0.001		0.065		0.422		<0.001		0.002		<0.001		0.006		<0.001		<0.001	
Season	0.229		0.956		0.092		0.711		0.475		0.626		0.174		0.034		0.8434	
Treatments x Season	0.295		0.092		0.259		0.126		0.061		0.0028		0.015		0.116		0.7654	

*Values followed by the same letter are not significantly different (Tuckey p ≤ 0.05). yield, Kg plant^-1^; N_cluster, number of cluster per plant; N_Grapes, number of grapes; W_Cluster, weight of cluster; V_Cluster, volume of cluster; W_Grape, weight of grape; D_Grape, diameter of grape; WP, water productivity (kg m^-3^); SI_Ψ_, integral water stress.*

The average WP was significantly higher in T3 than T1 and T2; the mean value in T3 was 7.3 kg m^-3^. Furthermore, T1 (4.06 kg m^-3^) and T2 (5.11 kg m^-3^) did not present significant differences between them. Regarding the seasonality analysis, compared with the 2011/12 season, the 2012/13 season showed a significantly higher mean value of 6.37 kg m^-3^ (**Table 7**). In this study, SS measured at harvest, showed significant differences among treatments in soluble solids for T2 (25.8°Brix) and T3 (25.7°Brix) compared to T1 (24.5°Brix) for the two seasons studied (data not shown). Finally, Supplementary Table S4 indicates a summary of an analysis of variance (ANOVA) expressed on the mean square and degree of freedom (df) for yield components, water productivity and integral water stress for both season studied.

**FIGURE 4:**
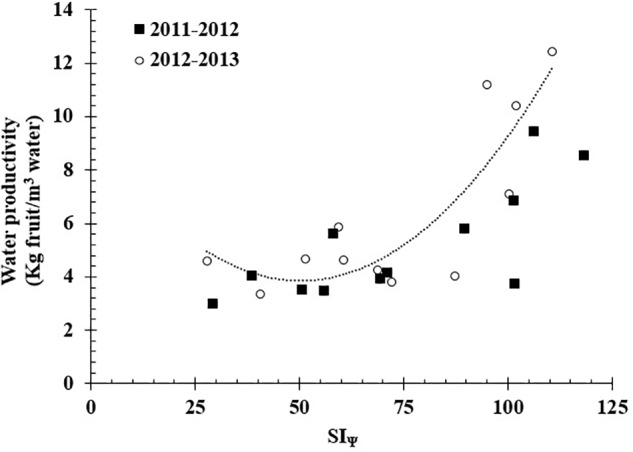
Relationship between integral water stress (SI_Ψ_) and water productivity (WP = 0.0022SI_Ψ_^2^–0.2206SI_Ψ_+9.396; *R*^2^ = 0.74) in a grafted Carménère vineyard during the 2011/12 and 2012/13 growing season.

The highest value of stem water potential (c) registered to calculate the SIΨ was -0.4 MPa for both studied seasons. The accumulative effect of water stress measured as SI_Ψ_ showed significant differences between all treatments; the highest mean values occurred for T3 (102.91 MPa), and the lowest mean values occurred for T1 (48.09 MPa). Between seasons, the SI_Ψ_ did not present significant differences. Finally, the SI_Ψ_ presented a significant non-linear relationship (*R*^2^ = 0.74) with WP (**Figure 4**).

## Discussion

The weather conditions during the two study seasons were in accordance with those of the expected climatic description of the area. The cumulative ETa of the grafted cv. Carménère (298–312 mm) observed in this study was close to that previously reported by Jara-Rojas et al. (2015) for an ungrafted cv. Carménère (315–349 mm) during B-H period. For this period, the values of GDD ranged between 1,473 and 1,547°C which were similar to those reported for other red cultivars. In this regard, Verdugo-Vásquez et al. (2016) reported a range between 1,455 and 1,640°C for Cabernet Sauvignon while Ramos et al. (2018) indicated 1,358 and 1,483°C for cvs. Cabernet Sauvignon and Tempranillo, respectively.

In this experiment, the water application for T2 and T3 was between 38.3–65.2% and 26.7–30.1% of the T1 treatment, respectively. In this case, the mean seasonal values of Ψ_s_ were -0.85, -1.1, and -1.3 MPa for T1, T2, and T3, respectively. The Ψ_s_ registered for the S-V period showed that T1 experienced a weak water deficit whereas T2 and T3 showed moderate water stress. During the V period, T1 showed moderate water stress, whereas T2 and T3 showed moderate to severe and severe water stress, respectively. For the V-H period, T1 showed moderate water stress, whereas both T2 and T3 showed moderate and severe water stress (**Tables 4**–**6**) (Sibille et al., 2007; Van Leeuwen et al., 2009). The most significant effects of water restriction were observed for the V period, which showed significant differences between the three irrigation strategies and presented the maximum level of stress in T3 (-1.50 MPa). The relatively low values of Ψ_s_ were similar to those reported by Intrigliolo et al. (2016) who reported values of approximately -1.6 MPa in rainfed treatments in cv. Cabernet Sauvignon (≈200 mm of rainfall during the season).

The maximum values of A_N_ observed in this study (14.0–19.0 μmol CO_2_ m^-2^s^-1^) are similar to those presented by Flexas et al. (1999) and Prieto et al. (2010), who reported maximum A_N_ values ranging between 20 and 21 μmol CO_2_ m^-2^s^-1^. However, these values are higher than those reported by other studies involving different cultivars, where maximum A_N_ values ranged between 15 and 16 μmol CO_2_ m^-2^s^-1^ (Romero et al., 2010; Poni et al., 2014; Jara-Rojas et al., 2015). In this case, mean values of A_N_ were between 64–75, 49–58, and 37–54% of A_Nmax_ for T1, T2, and T3, respectively. The maximum reduction of A_N_ was observed in T3 during V period.

Values of g_s_ ranged between 0.0 and 0.63 mol H_2_O m^-2^s^-1^ which are similar to those (0–0.5 mol H_2_O m^-2^s^-1^) reported by Flexas and Medrano (2002) for cv. Manto Negro and Tempranillo. However, g_s_ values of this study are higher than those reported by Romero et al. (2010), Prieto et al. (2010) and Chaves et al. (2007) who showed higher values of g_s_ between 0.25 and 0.4 mol H_2_O m^-2^s^-1^ for cv. Monastrell, Mourvèdre, Syrah, Marselan, Grenache, Ekigaïna, and Moscatel. In this study, mean values of g_s_ were significantly reduced by water stress from 0.28 to 0.16, 0.31 to 0.09, and 0.24 to 0.09 mol H_2_O m^-2^s^-1^ for the S-V, V and V-H periods, respectively.

Regarding reductions in photosynthesis due to stomatal closure, several authors have reported three phases of the photosynthetic response along a water stress gradient. In this case, g_s_ < 0.05, 0.05 < g_s_ < 0.15, and g_s_ > 0.15 mol H_2_O m^-2^s^-1^ are associated with severe, moderate and no water stress, respectively (Escalona et al., 1999; Flexas et al., 2002; Medrano et al., 2002; Cifre et al., 2005; Jara-Rojas et al., 2015). These ranges are in accordance with the piecewise linear regression analysis between A_N_/A_Nmax_ versus g_s_ observed in this study. However, the moderate and no water stress zones in this research showed differences in the thresholds and therefore in the range amplitude. Specifically, the upper level of the non-water stress conditions was for g_s_ > 0.30 mol m^-2^s^-1^, where an increase of g_s_ did not have a significant effect on the A_N_/A_Nmax_ trend (mean A_N_ was about 76% of A_Nmax_). Furthermore, moderate stress occurred within the interval 0.06 < g_s_ < 0.30 mol m^-2^s^-1^, which is wider than that reported in the literature. Moreover, the threshold of severe water stress was for g_s_ < 0.06 mol m^-2^s^-1^ which remained very close to previously reported values for grapevines (Escalona et al., 1999; Flexas et al., 2002; Medrano et al., 2002; Cifre et al., 2005; Jara-Rojas et al., 2015).

In this study, the relationship between Ψ_s_ and g_s_ presented a large scattering. This variability could be associated with instantaneous weather variable changes (Rs, Ws, and VPD) or with a specific physiological behavior linked to the cv. Carménère (Flexas and Medrano, 2002; Jara-Rojas et al., 2015). Additionally, the results of the data analysis showed that stomatal closure for the Carménère cv. could be not associated exclusively with hydraulic signals and processes. This finding is supported by the observed stomatal behavior which show that stomata can remain partially opened even when vines are subjected to severe water stress levels (Ψ_s_ = -1.5 MPa), with g_s_ values greater than 0.15 mol m^-2^s^-1^. These results are in accordance with those reported by Jara-Rojas et al. (2015), who classified the cv. Carménère as drought tolerant. This effect has been confirmed for other cultivars such as Shiraz, in which applying restricted irrigation strategies resulted in increased WUE primarily via increased stomatal sensitivity to both water loss and VPD triggered by root-to-shoot hormone signaling, such as that involving abscisic acid (Collins et al., 2010).

The yield component analysis showed results similar to those of other RDI studies on grapevines. Specifically, the total yield was affected mainly because water stress reduced berry diameter and weight. Since there were no significant differences between T2 and T3, it is expected that grape size was affected mainly during the earliest stage of grape development (Acevedo-Opazo et al., 2010; Basile et al., 2011). Comparable results were reported by Santesteban et al. (2011) who observed that water stress at the beginning of berry development resulted in important reductions in berry weight. These reductions were not increased by applications of water stress in the period between V and H. In contrast to the results reported in this study, Chaves et al. (2007) did not report significant differences in yield between treatments irrigated at 100 and 50% of ET_a_ in cv. Castelao and Moscatel. In the same way, Trigo-Cordoba et al. (2015) reported no differences in yield for the cv. Godello under rainfed conditions and irrigation at 50% of ET_a_. An explanation for these differences is related to levels of the water stress reached by the vines. The restricted irrigation treatments done by Chaves et al. (2007) and Trigo-Cordoba et al. (2015) only reached out a moderate water stress instead in our experiment Carménère vines were under moderate to severe water stress.

Similar to this study, Intrigliolo et al. (2016) found for cv. Cabernet Sauvignon that there were significant differences between rainfed conditions and irrigation at 75% of ET_a_ during the V-H period, with yields of 1.9 and 2.4 kg plant^-1^, respectively. In this experiment, the WP was 2.7 kg m^-3^ under rainfed conditions and 1.7 kg m^-3^ at 75% of ET_a_. These values of WP are lower than those observed in the present study, which were 4.0 and 7.3 kg m^-3^ for T1 and T3, respectively. These differences were strongly influenced by the amount of rainfall, which in the study reported by Intrigliolo et al. (2016) was approximately 150 mm higher during the growing season than the present study.

The SI_Ψ_ appeared to be an appropriate parameter for evaluating the effects of water deficit and its relationship with water productivity but did not show a good performance in terms of yield parameters (Zuñiga et al., 2017).

Although small decreases in the plant water potential could cause stomatal closure and a decrease in the intensity of CO_2_ assimilation, there are several physiological and molecular mechanisms that differentiate the level of the damage caused by water stress. Diverse studies carried out in different “cultivasr” have shown that there is a differential expression of genes involved in photosynthesis and gas exchange process which are expressed when plants are affected by different abiotic stress such as: water stress, salt, iron, cold and even light quality (Do Amaral et al., 2016; Liu et al., 2016; Saha et al., 2016; Vineeth et al., 2016). These abiotic stress directly affect on WUE through the gene expression linked to Rubisco, photorespiration process and photosystems functioning. The gene expression presents a high variability among species and also within the same species. In this sense, Tortosa et al. (2016) in a study carried out on 23 cultivars (red and white) and 30 genotypes of Tempranillo found that there was a very high variability in WUE_i_ with coefficients of variation (CV) ranging between 26 and 32%, respectively.

Finally, future research will evaluate the effect of RDI on grape and wine quality of cv. Carménère grafted on different rootstocks. This issue is very important because the commercial harvest of this cultivar is determined by the phenolic maturity (minimum seed tannins, maximum anthocyanins) (Fredes et al., 2010) to minimize the perception of astringency and is not related to a specific soluble solids content.

## Conclusion

The irrigation strategies implemented in this study had a significant effect on gas exchange variables, WUEi, WUE, WP and yield components in grafted Carménère grapevines. The physiological variables analyzed in this study were altered by the irrigation strategies and showed significant differences during the period between veraison and harvest. Number of clusters and number of berries were not affected by the irrigation strategies; however, the rest of the yield components were affected, with a significant reduction in yield per plant. In terms of water productivity, T3 presented the highest value (7.3 kg m^-3^), with a clear compensation effect regarding the use of less water (reduction in irrigation water of approximately 70%) to support the yield. These results were consistent in both study seasons. In addition, it was possible to obtain a non-linear relationship between SI_Ψ_ and WP. The results indicated that for improving WUE without affecting yield is recommended to apply RDI from veraison period. The evidence indicates that Carménère is tolerant to water stress and that it could be an appropriate cultivar to consider under severe water stress conditions in future climate change scenarios. Further research is necessary to evaluate the response of cv. Carménère grafted on different rootstocks to water stress conditions.

## Author Contributions

SO-F and MZ conceived and designed the experiments. MZ performed the evaluations. SO-F, MZ, CR-B, SF, and CP-E analyzed the data and wrote the paper.

## Conflict of Interest Statement

The authors declare that the research was conducted in the absence of any commercial or financial relationships that could be construed as a potential conflict of interest.
