# Supplementary material for: Effects of Three Irrigation Strategies on Gas Exchange Relationships, Plant Water Status, Yield Components and Water Productivity on Grafted Carménère Grapevines
Source: Front Plant Sci. 2018 Jul 12;9:992. doi: 10.3389/fpls.2018.00992 (PMC6052738; doi:10.3389/fpls.2018.00992)
Supplement: Supplementary file 1 [file Table_1.pdf]

Supplementary information

Table S1. Summary of analysis of variance (ANOVA) expressed on the mean square and degree of freedom (df) for physiological variables from setting (S) to veraison (V) period of a drip-irrigated Carménère vineyard.

Source	df	Ψ_s	A_N/A_{Nmax}	g_s/g_{smax}	E/E_{max}	WUE_i	WUE
Treatment	2	1.39	18	0.18	0.12	387.16	0.004
Season	1	2.23	0.05	0.05	1.11	888.74	1.77
Treatment x season	2	0.2	0.18	0.18	0.16	546.66	0.03
Error	42	0.04	0.02	0.02	0.02	115.92	0.07

Table S2. Summary of analysis of variance (ANOVA) expressed on the mean square and degree of freedom (df) for physiological variables during veraison (V) period of a drip-irrigated Carménère vineyard.

Source	df	Ψ_s	AN/ANmax	gs/gsmax	E/Emax	WUEi	WUE
Treatment	2	2.8	1.09	1.44	1.04	4948.48	0.44
Season	1	0.02	0.2	0.11	0.61	4412.1	6.71
Treatment x season	2	0.36	0.02	0.01	0.01	71.54	0.07
Error	78	0.06	0.02	0.03	0.02	167.81	0.14

Table S3. Summary of analysis of variance (ANOVA) expressed on the mean square and degree of freedom (df) for physiological variables from veraison (V) to harvest (H) period of a drip-irrigated Carménère vineyard.

Source	df	Ψ_s	A_N/A_{Nmax}	g_s/g_{smax}	E/E_{max}	WUE_i	WUE
Treatment	2	1.95	0.61	0.79	0.70	4519.98	1.05
Season	1	0.96	0.65	0.11	0.22	7451.99	0.25
Treatment x season	2	0.35	0.09	0.14	0.13	444.10	0.03
error	131	0.07	0.02	0.03	0.02	329.49	0.19

Table S4. Summary of analysis of variance (ANOVA) expressed on the mean square and degree of freedom (df) for yield components, water productivity and integral water stress for both season studied.

Source	df	yield	N_cluster	N_Grapes	W_Cluster	V_Cluster	W_Grape	D_Grape	WP	SI_{Ψ}
Treatment	2	2.09	13.63	181.84	3852.21	4962.21	0.4	4.2	61.37	6098.96
Season	1	0.2	0.01	645.84	48.51	282.22	0.01	1.19	8.64	9.88
Treatment x Season	2	0.17	11.27	292.05	800.47	2539.6	0.31	3.16	3.99	66.78
Error	18	0.13	3.29	200.71	343.39	529.15	0.04	0.6	1.65	246.04

yield= Kg $plant^{-1}$; N_cluster= number of cluster per plant; N_Grapes= number of grapes; W_Cluster= weight of cluster; V_Cluster= volume of cluster; W_Grape= weight of grape; D_Grape= diameter of grape; WP= water productivity (kg m^{-3}); SI_{Ψ}= integral water stress.
